# Comparative chloroplast genomics clarifies the taxonomic status and supports molecular identification of *Viburnum keteleeri* ‘Sterile’ and *Viburnum keteleeri*

**DOI:** 10.3389/fpls.2026.1908462

**Published:** 2026-07-28

**Authors:** Yanlei Liu, Xin Shen, Junqi Liang, Liyuan Tian, Ye Huang, Weili Tian, Fei Zhuge, Dongyue Jiang

**Affiliations:** 1 School of Landscape and Ecological Engineering, Hebei University of Engineering, Handan, China; 2 Institute of Tree Breeding, Zhejiang Academy of Forestry, Hangzhou, China; 3 Hangzhou Botanical Garden, Hangzhou West Lake Academy of Landscape Science, Hangzhou, China; 4 Hangzhou Landscaping Incorporated, Hangzhou, China

**Keywords:** chloroplast genome, taxonomic status, phylogenetic analysis, species identification, *Viburnum keteleeri*, *Viburnum keteleeri* ‘Sterile’

## Abstract

*Viburnum keteleeri* ‘Sterile’ and *V. keteleeri* are traditional ornamental plants in China and are highly valued for their large, spherical inflorescences. However, due to long-term artificial selection and morphological similarity, the taxonomic boundary between *V. keteleeri* ‘Sterile’ and *V. keteleeri* has remained controversial. Conventional morphological identification and conventional barcodes have proven insufficient for accurately resolving their taxonomic relationship. In this study, *V. keteleeri* ‘Sterile’, *V. keteleeri*, and their closely related species were investigated using complete chloroplast genome (CPG) data. Comparative genomic analyses were subsequently performed using additional CPGs of *Viburnum* species retrieved from public databases. The results showed that both CPGs exhibited the typical quadripartite structure of angiosperms, each with a total length of 158,662 bp and containing 133 annotated genes. Simple sequence repeat analysis revealed that T/A-type repeats were the most abundant SSR category, while codon usage analysis indicated a strong preference for synonymous codons ending in A/U. Comparative analyses of 49 *Viburnum* CPGs demonstrated extremely low genetic divergence between *V. keteleeri* ‘Sterile’ and *V. keteleeri*. Phylogenetic reconstruction consistently placed the two taxa within the same terminal clade, suggesting a very close relationship. Based on the available chloroplast genome evidence, *V. keteleeri* ‘Sterile’ is unlikely to represent the nominal species and is more appropriately regarded as a horticultural cultivar or cultivated form derived from *V. keteleeri*. In addition, 293 lineage-specific single nucleotide polymorphism (SNP) loci were identified, and the *ycf1* genes of both *V. keteleeri* ‘Sterile’ and *V. keteleeri* were found to be significantly longer than those of the other examined *Viburnum* species. These genomic features provide valuable candidate markers for rapid and accurate molecular identification. Overall, this study provides valuable genomic resources for clarifying taxonomic relationships, supporting germplasm conservation, facilitating cultivar authentication, and strengthening plant variety protection and nursery stock quality control in *Viburnum*.

## 1 Introduction

*Viburnum keteleeri* (Qionghua) and its sterile-flowered horticultural form, *V. keteleeri* ‘Sterile’ (Chinese snowball viburnum), are among the most iconic ornamental shrubs native to China (Jin et al., 2010; Editorial Committee of the Flora of China CAoS, 2011; Lu et al., 2017; Ran et al., 2020). In this study, we follow the taxonomic treatment adopted in the Flora Reipublicae Popularis Sinicae, in which Qionghua is recognized as *Viburnum keteleeri* at the species level rather than as *V. macrocephalum f. keteleeri*. Accordingly, the sterile-flowered horticultural form is treated as *V. keteleeri* ‘Sterile’. Owing to their showy white inflorescences, attractive growth habit, and broad environmental adaptability, both taxa have been cultivated for centuries in traditional Chinese gardens and have become important ornamental plants worldwide since their introduction to Europe in the nineteenth century. Chinese snowball viburnum, in particular, is highly valued in horticulture because its inflorescences consist entirely of enlarged sterile flowers that form conspicuous globose flower clusters resembling hydrangea inflorescences and snowballs-like structures (Jin et al., 2010; Editorial Committee of the Flora of China CAoS, 2011; Lu et al., 2017; Ran et al., 2020).

Despite their horticultural importance, the taxonomic relationship between *V. keteleeri* and *V. keteleeri* ‘Sterile’ has long been controversial. Traditional taxonomic treatments recognized Chinese snowball viburnum as the nominal species, *V. keteleeri* ‘Sterile’, whereas Qionghua was treated as *V. keteleeri* (Flora Reipublicae Popularis Sinicae, Flora of China). Such classifications were largely based on differences in inflorescence architecture and floral fertility (Editorial Committee of the Flora of China CAoS, 2011). However, morphological traits associated with ornamental value are often influenced by artificial selection and domestication, which may obscure evolutionary relationships and reduce their utility for species delimitation (Duminil and Di Michele, 2009; Gregory, 2009; Fuller et al., 2014; Altman et al., 2022). Developmental studies have shown that the enlarged sterile flowers of Qionghua and related ornamental forms evolved primarily through modifications of floral organ identity and function, suggesting that these characteristics may represent derived horticultural characteristics rather than species-level differences (Jin et al., 2010; Editorial Committee of the Flora of China CAoS, 2011; Lu et al., 2017; Ran et al., 2020).

Recent taxonomic revisions and evolutionary studies have increasingly supported the view that Chinese snowball viburnum originated through long-term horticultural selection from *V. keteleeri* (Qionghua) rather than representing an independently evolved wild species (Editorial Committee of the Flora of China CAoS, 2011). Nevertheless, the taxonomic status of this sterile-flowered form remains inadequately supported by genome-scale molecular evidence (Clement and Donoghue, 2012). Accurate delimitation of these taxa is important not only for taxonomic classification but also for germplasm conservation, cultivar registration, breeding programs, and intellectual property protection in ornamental horticulture.

Molecular markers have become indispensable tools for plant identification and phylogenetic inference (Hebert et al., 2003; Kress and Erickson, 2007; Hollingsworth et al., 2009; Dong et al., 2015; Li et al., 2015; Kress, 2017). However, studies of DNA barcoding in *Viburnum* have revealed that species discrimination decreases substantially among closely related taxa because of limited sequence divergence in standard barcode regions, including *rbcL*, *matK*, and nuclear ITS (internal transcribed spacer region). In a comprehensive evaluation of 112 *Viburnum* species, Clement and Donoghue (2012) demonstrated that commonly used barcode loci possess relatively low discriminatory power within the genus, highlighting the difficulty of resolving recent divergences using a few short molecular markers alone (Clement and Donoghue, 2012; Li et al., 2015). Consequently, higher-resolution genomic resources are required to clarify relationships among closely related taxa and cultivated derivatives.

The rapid development of next-generation sequencing (NGS) technology has promoted the widespread application of complete chloroplast genomes (CPGs) in plant systematics (Metzker, 2010; Li et al., 2015; The Angiosperm Phylogeny Group et al., 2016; Zuntini et al., 2024). Compared with conventional DNA barcodes, CPGs provide substantially more phylogenetically informative characters and have therefore been proposed as “super-barcodes” for species identification and phylogenetic reconstruction (Li et al., 2015). Owing to their conserved structure, predominantly uniparental inheritance, and moderate evolutionary rate, chloroplast genomes have proven particularly effective for resolving relationships among closely related species and identifying lineage-specific molecular markers (Dong et al., 2022; Liu et al., 2025b; Liu et al., 2025a; Li et al., 2026). Recent comparative CPG studies within Viburnaceae have further demonstrated the utility of chloroplast genomes for phylogenetic reconstruction, divergence analysis, and molecular marker development (Ran et al., 2020).

In addition, comparative chloroplast genomics has become an important component of horticultural multi-omics research. Recent studies have shown that CPG analyses can effectively identify highly variable regions, simple sequence repeats (SSRs), and diagnostic single nucleotide polymorphisms (SNPs), thereby facilitating cultivar identification, germplasm evaluation, and phylogenomic analyses in ornamental plants (Dong et al., 2022; Liu et al., 2025b; Liu et al., 2025a; Li et al., 2026). Such approaches provide unprecedented opportunities to investigate the evolutionary origin and domestication history of horticultural taxa.

Therefore, in the present study, we sequenced and compared the CPGs of *V. keteleeri* and *V. keteleeri* ‘Sterile’. Through comparative CPG analyses, identification of highly variable regions and lineage-specific SNPs, and phylogenetic reconstruction based on CPGs, we aimed to (i) provide support of clarifying the taxonomic status of Chinese snowball viburnum and Qionghua, (ii) evaluate the effectiveness of chloroplast genomes for distinguishing the sterile cultivar from its putative ancestral species, and (iii) develop molecular resources for accurate identification and germplasm management. The results will provide new insights into the domestication and evolutionary history of this important ornamental plant, while contributing valuable genomic resources for breeding, conservation, and horticultural utilization.

## 2 Materials and methods

### 2.1 Plant materials and chloroplast genome dataset

This study aimed to evaluate the taxonomic status and species identification efficiency of *V. keteleeri* ‘Sterile’ and *V. keteleeri* using CPGs as super-barcodes. Fresh leaf samples of *V. keteleeri* ‘Sterile’ and its putative progenitor species, *V. keteleeri*, were collected for chloroplast genome sequencing. Fresh plant materials were collected from Hangzhou Botanical Garden, China. The species identities were determined by Dr. Dongyue Jiang based on the morphological characters and identification criteria described in Flora Reipublicae Popularis Sinicae. Voucher specimens were deposited in the herbarium of the Institute of Tree Breeding, Zhejiang Academy of Forestry, under the voucher numbers JDYVD001 and JDYVD003. Additional chloroplast genome sequences representing major lineages of *Viburnum* and related taxa were retrieved from the NCBI GenBank database. Species of *Sambucus* were selected as outgroups for phylogenetic analyses. All CPGs were integrated into a unified dataset for comparative genomic and phylogenetic analyses.

### 2.2 Chloroplast genome sequencing, assembly, and annotation

#### 2.2.1 DNA extraction and sequencing

Total genomic DNA was extracted from silica-gel-dried leaves using a modified CTAB method (Li et al., 2013). DNA quality and concentration were evaluated using agarose gel electrophoresis and fluorometric quantification. Approximately 1 μg of high-quality genomic DNA was fragmented to an average size of 350 bp using a Covaris ultrasonicator (Covaris, Woburn, MA, USA). Sequencing libraries were constructed following standard Illumina protocols, including end repair, A-tailing, adapter ligation, and PCR enrichment. Library quality was assessed using an Agilent 2100 Bioanalyzer and quantified by qPCR. Paired-end sequencing (PE150) was performed on the Illumina NovaSeq 6000 platform, generating approximately 5 Gb of raw data per sample.

#### 2.2.2 Genome assembly and annotation

Raw reads were filtered using Trimmomatic v0.39 to remove adapter sequences (Bolger et al., 2014), low-quality reads (Q < 20), and reads shorter than 36 bp. The resulting clean reads were assembled *de novo* using NOVOPlasty v4.3.1, with the chloroplast *rbcL* gene from a closely related species used as the seed sequence (Dierckxsens et al., 2017). Initial genome annotation was conducted using the CPGAVAS2 online platform (Shi et al., 2019). The annotation results were subsequently verified and refined using PGA, and manually curated in Geneious Prime 2021 to ensure accurate gene boundaries, start/stop codons, and intron-exon structures (Kearse et al., 2012; Qu et al., 2019). Circular chloroplast genome maps were generated using OGDRAW integrated within CPGAVAS2 (Shi et al., 2019). CPGView (http://www.1kmpg.cn/cpgview/) and PlastidHub (https://www.plastidhub.cn/utils) were also used for data visualization in this study.

### 2.3 Comparative chloroplast genome analyses

#### 2.3.1 Whole-genome sequence comparison

Complete chloroplast genomes of *Viburnum* species were compared using the mVISTA program under the Shuffle-LAGAN alignment mode (Frazer et al., 2004). The chloroplast genome of *V. keteleeri* ‘Sterile’ was used as the reference sequence to visualize sequence conservation and divergence among coding and non-coding regions.

#### 2.3.2 Nucleotide diversity analysis

Multiple sequence alignments were generated using MAFFT v7.409 (Katoh and Standley, 2013). Nucleotide diversity (Pi) was calculated using DnaSP v6 with a sliding-window approach (Rozas et al., 2017). The window length and step size were set to 600 bp and 200 bp, respectively. Regions with high nucleotide variability (Pi > 0.02) were identified as potential molecular markers.

#### 2.3.3 Structural and sequence variation analyses

Structural variations among chloroplast genomes were investigated using IRplus by examining expansion and contraction events at the LSC/IR and SSC/IR junctions (Díez Menendez et al., 2023). Particular attention was paid to the distribution of boundary genes such as *rpl19* and *ycf1*. Sequence variation patterns were further evaluated through whole-genome alignments generated by mVISTA to identify highly divergent genomic regions (Frazer et al., 2004).

### 2.4 Phylogenetic analyses

The sequences were aligned using MAFFT v7.313 with an auto strategy and then trimmed by trimAl v1.2 (Capella-Gutiérrez et al., 2009; Katoh and Standley, 2013). The best-fit models for maximum-likelihood (ML) analyses were selected according to the Bayesian information criterion (BIC) by ModelFinder (Kalyaanamoorthy et al., 2017). The ML method was used to construct the phylogenetic tree. ML analyses were performed using IQ-TREE v.1.6.8 (Minh et al., 2020), with the GTR+F+I model and 1000 standard bootstrap replicates. The phylogenetic trees and branch support values were visualized using FigTree v.1.4.2.

### 2.5 Species identification based on chloroplast genome sequences

#### 2.5.1 Species identification using chloroplast super-barcodes

CPGs of *V. keteleeri* ‘Sterile’, *V. keteleeri*, and representative *Viburnum* species were used as super-barcodes. Phylogenetic analyses based on CPG alignments were conducted to evaluate species discrimination power and determine the taxonomic placement of *V. keteleeri* ‘Sterile’ and *V. keteleeri*.

#### 2.5.2 Development of molecular markers

To develop practical markers for rapid species identification, lineage-specific single nucleotide polymorphisms (SNPs) and insertion/deletion polymorphisms (InDels) were identified through comparative chloroplast genome analyses. Highly variable regions containing diagnostic SNPs and InDels were selected as candidate loci for marker development. These loci may serve as targets for designing lineage-specific primers or universal amplification primers suitable for high-throughput amplicon sequencing, thereby enabling accurate discrimination of *V. keteleeri* ‘Sterile’ and *V. keteleeri* from closely related *Viburnum* taxa.

## 3 Results

### 3.1 Chloroplast genome features of *V. keteleeri* ‘sterile’ and *V. keteleeri*

**Figure 1:**
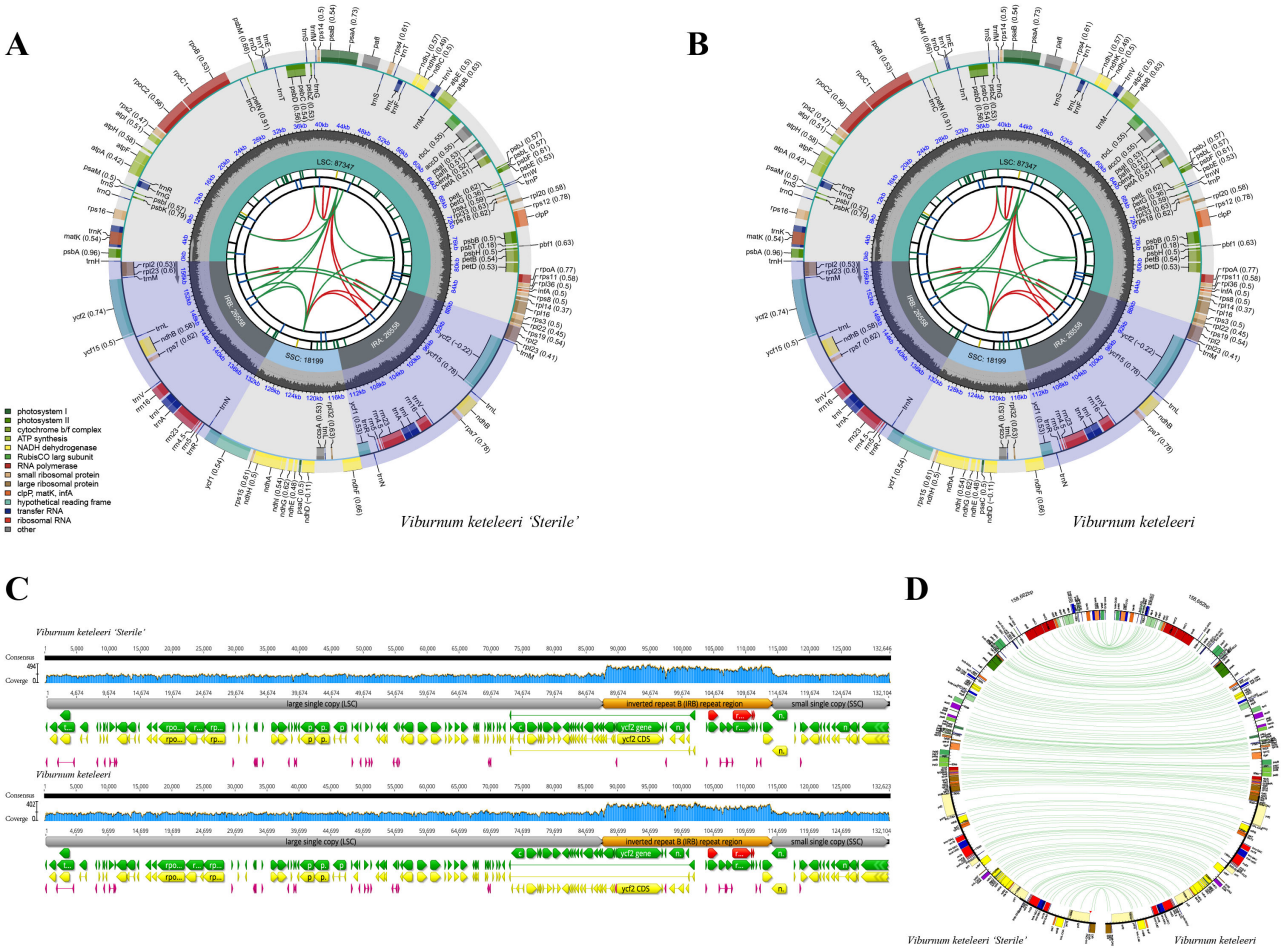
V*. keteleeri* ‘Sterile’ and *V. keteleeri* chloroplast genome features. **(A)** Physical map of the chloroplast genome of *V. keteleeri* ‘Sterile’; **(B)** Physical map of the chloroplast genome of *V. keteleeri*; **(C)** Sequencing depth across the chloroplast genomes of *V. keteleeri* ‘Sterile’ and *V. keteleeri*; **(D)** Comparative analysis of gene order in the chloroplast genomes of *V. keteleeri* ‘Sterile’ and *V. keteleeri*.

The complete chloroplast genome of *V. keteleeri* ‘Sterile’ and *V. keteleeri* exhibited a typical quadripartite structure and was identical in length (158,662 bp) (**Figures 1A, B**) and differed by only a single nucleotide (**Supplementary Figure 1**). Both assembled chloroplast genomes exhibited an average sequencing depth greater than 100×, while the maximum coverage within the IR regions exceeded 400×. Notably, the sequencing depth of the IR regions was nearly double that of the large single-copy (LSC) and small single-copy (SSC) regions, reflecting the presence of two identical IR copies in the chloroplast genome (**Figure 1C**). The genomes of the two CPGs consisted of a large single-copy (LSC) region of 87,347 bp, a small single-copy (SSC) region of 18,199 bp, and a pair of inverted repeat (IR) regions of 26,558 bp each. A total of 133 genes were annotated, including 86 protein-coding genes, 37 tRNA genes, and eight rRNA genes. The overall gene composition and genome organization were highly conserved and consistent with those reported for other species of *Viburnum* (**Figures 1A, B**). The two assembled chloroplast genomes were completely identical in genome structure and gene order, indicating a high degree of structural conservation between the two taxa (**Figure 1D**).

The junctions between the IR and single-copy regions were occupied by genes including *ycf1*, *rps19*, and *ndhF*. Variation in the positions of these boundary genes was observed among *Viburnum* species, reflecting differences in IR expansion and contraction events (**Supplementary Figure 2**).

### 3.2 Comparative analysis of chloroplast genomes

#### 3.2.1 Identification of highly variable regions

**Figure 2:**
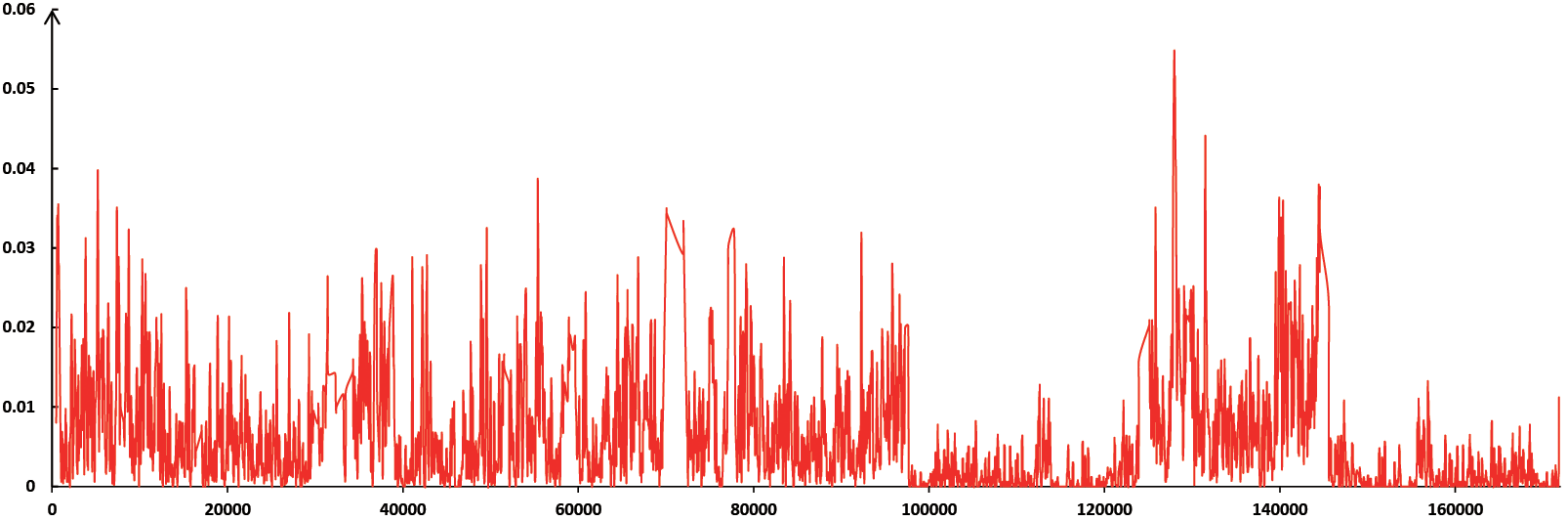
Nucleotide diversity (Pi) across the chloroplast genomes of *Viburnum* species. The x-axis indicates nucleotide positions in the multiple-sequence alignment, whereas the y-axis represents nucleotide diversity (Pi) estimated using a sliding-window analysis.

Comparative genomic analyses revealed that non-coding regions exhibited substantially higher sequence divergence than coding regions, whereas the IR regions were highly conserved (**Figure 2**). Nucleotide diversity (Pi) values across the chloroplast genomes showed marked heterogeneity. The lowest Pi values were detected within the IR regions, particularly between approximately 100 kb and 120 kb, where nucleotide diversity was close to zero. In contrast, highly variable regions were mainly distributed in the LSC and SSC regions, with maximum Pi values approaching 0.06.

#### 3.2.2 Distribution of simple sequence repeats (SSRs)

**Figure 3:**
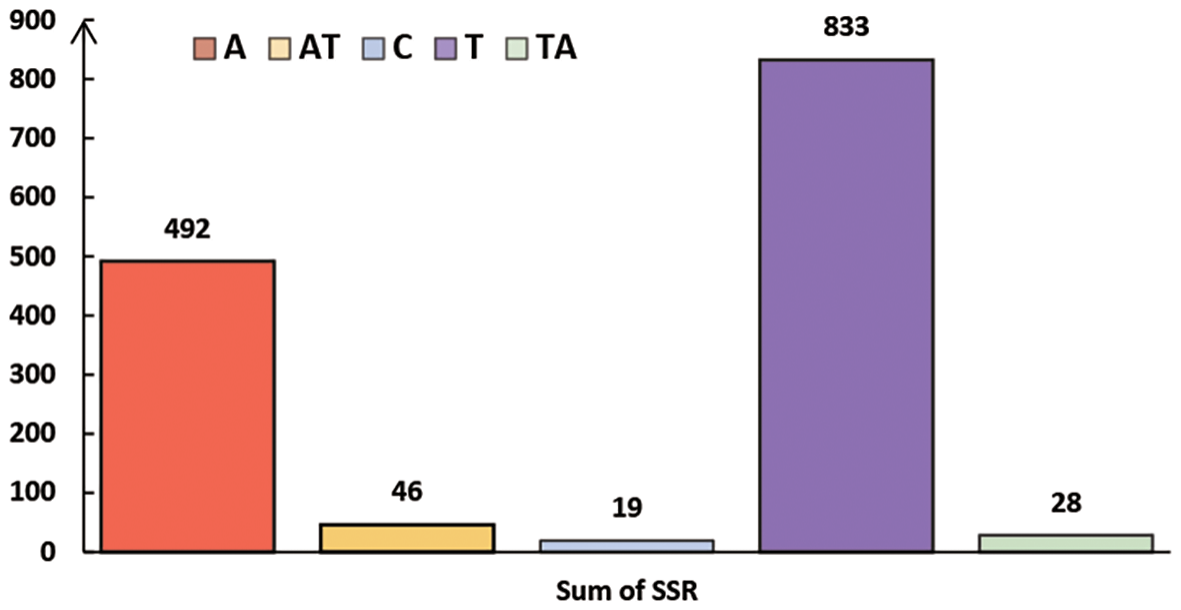
Distribution of simple sequence repeats (SSRs) in the chloroplast genomes of *V. keteleeri* ‘Sterile’ and *V. keteleeri*.

A total of 1,418 SSR loci were identified in the chloroplast genome of *V. keteleeri* ‘Sterile’ and *V. keteleeri* (**Figure 3**). Mononucleotide repeats represented the predominant repeat type, accounting for the majority of SSRs detected. Among them, T-type repeats were the most abundant (833 loci), followed by A-type repeats (492 loci). Dinucleotide repeats were relatively rare, with only 46 AT repeats and 28 TA repeats identified. These results indicate a strong A/T-rich composition within the chloroplast genome.

#### 3.2.3 Intron-containing genes and codon usage bias

**Figure 4:**
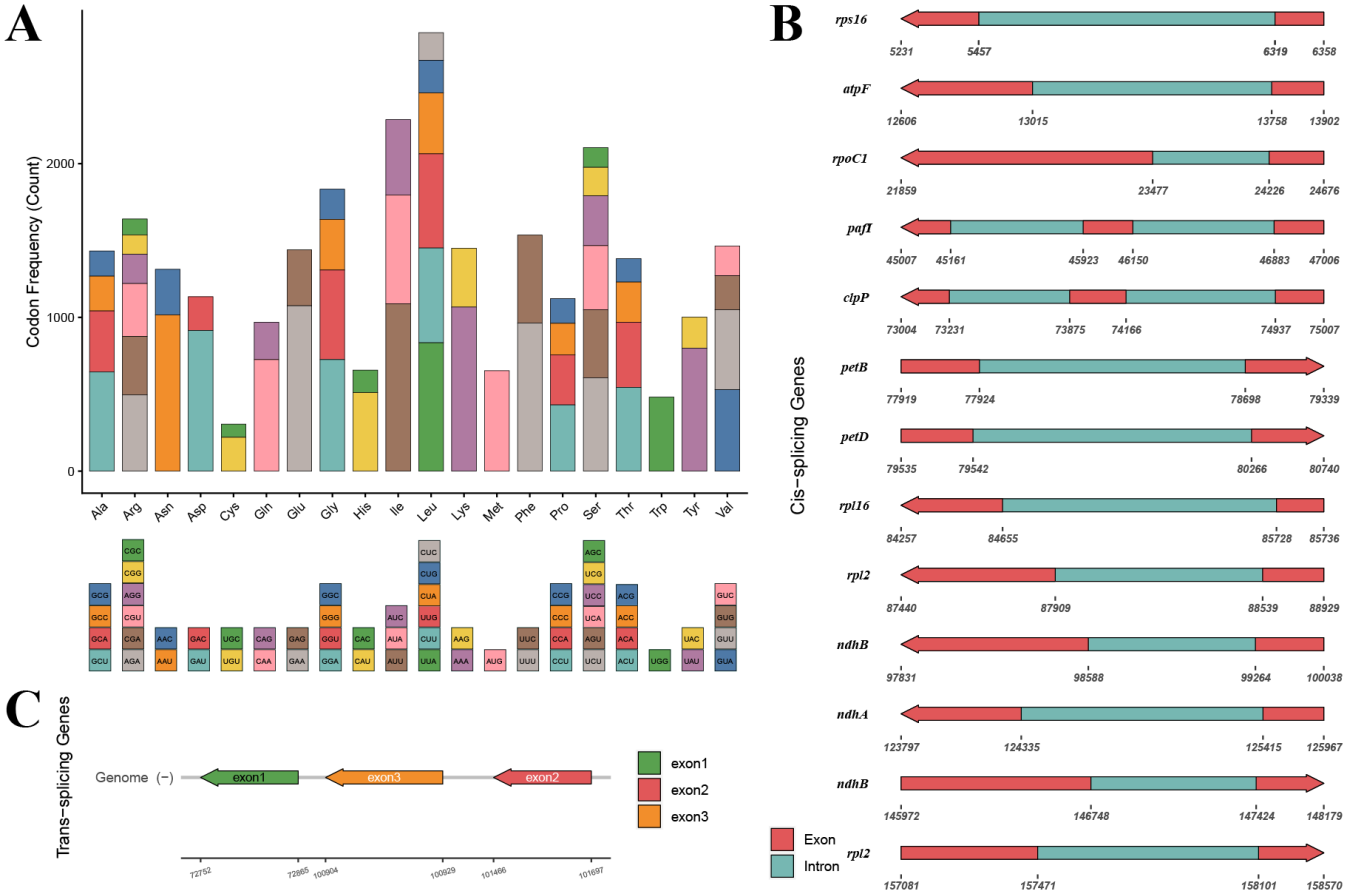
Codon usage bias and cis-/trans-splicing gene organization in the chloroplast genome of *V. keteleeri* ‘Sterile’ and *V. keteleeri*. **(A)** Codon usage frequencies of chloroplast protein-coding genes; **(B)** Exon-intron structures of cis-spliced genes; **(C)** Exon organization of the trans-spliced gene *rps12*. Boxes indicate exons and lines indicate introns. The positions of exons in different genomic regions are connected through trans-splicing to form a mature transcript.

Analysis of synonymous codon usage revealed obvious codon usage bias (**Figure 4A**). Leucine, isoleucine, serine, glycine, and valine were the most frequently encoded amino acids, whereas cysteine and tryptophan occurred least frequently. Most preferred codons ended with A or U, indicating a pronounced A/T bias in the coding sequences of the chloroplast genome. A total of 18 intron-containing genes were identified in the chloroplast genome (**Figures 4B, C**). Most genes contained a single intron, whereas *clpP* and *ycf3* contained two introns. The trans-spliced gene *rps12* exhibited a typical trans-splicing structure, with the first exon located in the LSC region and the remaining exons duplicated within the IR regions.

### 3.3 Phylogenetic relationships of *V. keteleeri* ‘Sterile’ and *V. keteleeri*

**Figure 5:**
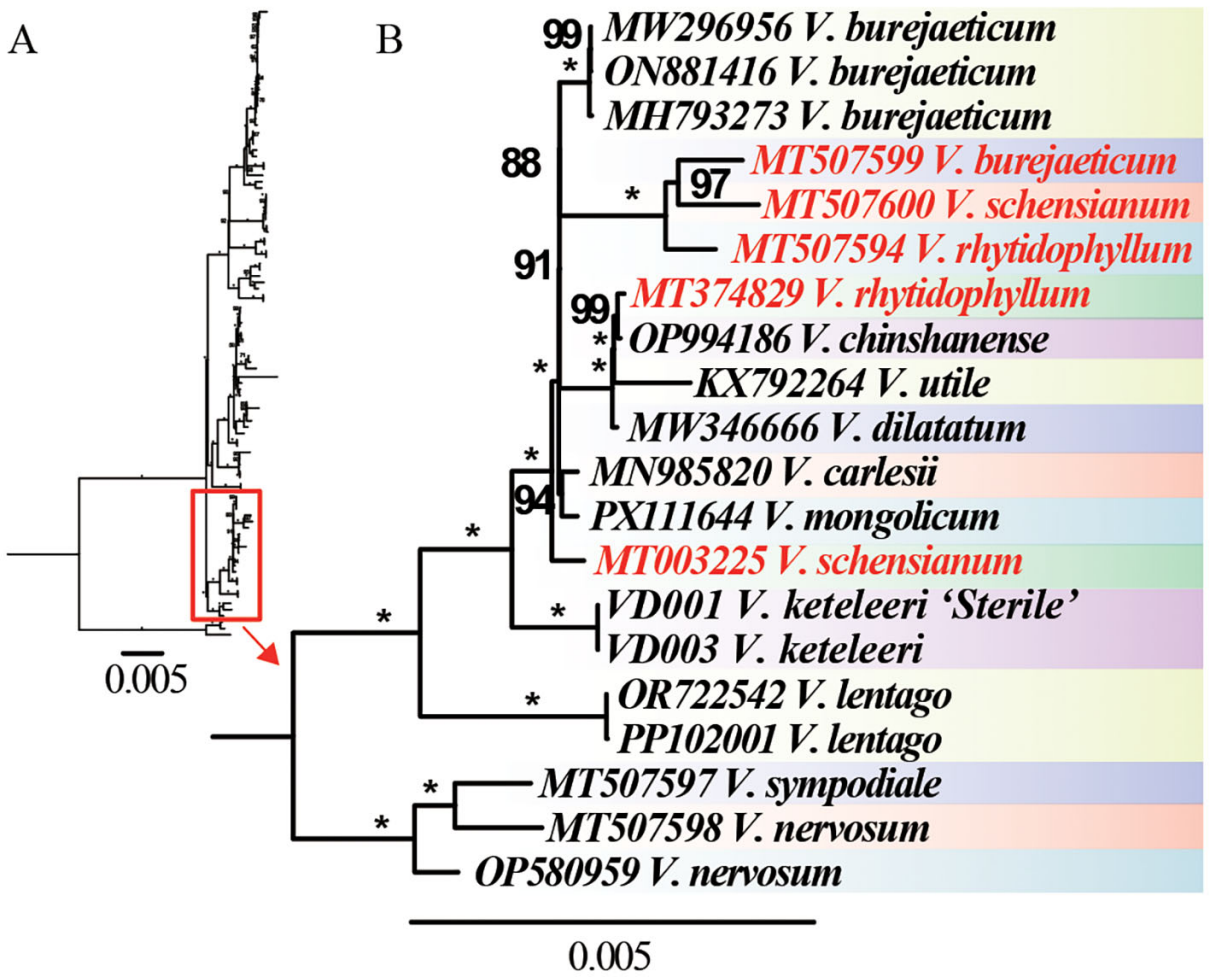
Phylogenetic relationships among *Viburnum* species inferred from complete chloroplast genome sequences. **(A)** ML phylogenetic framework of *Viburnum* inferred from currently available complete chloroplast genome sequences, showing the overall evolutionary relationships among sampled taxa. **(B)** Detailed ML phylogenetic relationships of the focal clade investigated in this study, including *Viburnum keteleeri* and *Viburnum keteleeri* ‘Sterile’. Bootstrap values are indicated at the nodes, and an asterisk (*) denotes 100% bootstrap support.

Phylogenetic analysis based on CPGs generated a well-resolved topology with strong branch support (**Figure 5**). *V. keteleeri* ‘Sterile’ clustered with *V. keteleeri* and formed a highly supported monophyletic lineage. The extremely short branch length separating the two taxa indicated minimal chloroplast genome divergence. In contrast, this lineage was clearly separated from other *Viburnum* species, demonstrating the effectiveness of complete chloroplast genomes in resolving interspecific relationships within the genus. Furthermore, we observed that multiple accessions of the same species obtained from public databases did not consistently cluster into a single monophyletic clade. For example, samples of *V. burejaeticum*, *V. rhytidophyllum*, and *V. schensianum* were distributed across different branches of the phylogenetic tree rather than grouping together as expected (**Figure 5**). This phylogenetic incongruence may result from specimen misidentification, interspecific hybridization, chloroplast capture, or unresolved species boundaries, and warrants further investigation using nuclear genomic data and morphological evidence.

### 3.4 Species identification based on chloroplast genome variation

Because only a single nucleotide difference (cultivar-discriminating SNP) was detected between V*. keteleeri* ‘Sterile’ and *V. keteleeri*, only the *V. keteleeri* sample was retained for subsequent structural analyses (**Supplementary Figure 1**). Although only one nucleotide difference was detected between the plastomes of *V. keteleeri* and *V. keteleeri* ‘Sterile’, we further investigated whether this variation represented a stable lineage-associated polymorphism rather than an accession-specific mutation. To address this concern, additional samples from independent individuals of *V. keteleeri* and *V. keteleeri* ‘Sterile’ maintained at Hangzhou Botanical Garden were analyzed. Based on the plastome comparison, we hypothesized that the diagnostic nucleotide position would exhibit distinct chromatogram patterns between the two horticultural lineages. Specifically, *V. keteleeri* showed an A/C polymorphism at this position, and therefore we expected chromatograms of *V. keteleeri* individuals to display two nucleotide peaks, with A as the predominant signal and C as the secondary signal. In contrast, *V. keteleeri* ‘Sterile’ was expected to exhibit a dominant A peak without a comparable secondary C signal. The validation results were consistent with this hypothesis. After excluding the dominant A signal, the C peak represented the second strongest nucleotide signal in all three analyzed individuals of *V. keteleeri* (**Supplementary Figure 3, Samples 1–3**). In contrast, the three individuals of *V. keteleeri* ‘Sterile’ did not exhibit a comparable secondary nucleotide peak pattern (**Supplementary Figure 3, Samples 4–6**). These results indicate that the identified chloroplast nucleotide variation is consistently associated with the two horticultural lineages and is unlikely to represent an individual-specific polymorphism from the original plastome assembly. Although additional nuclear genomic evidence will be required to further clarify the evolutionary origin and genetic basis of the sterile-flower phenotype, this stable chloroplast variation provides a useful diagnostic marker for distinguishing *V. keteleeri* from *V. keteleeri* ‘Sterile’.

Complete chloroplast genome alignments of more than 30 *Viburnum* species revealed a highly conserved gene order and genome organization across the genus (**Supplementary Figure 2**). However, several distinct structural features were observed in *V. keteleeri* ‘Sterile’. The *ycf1* gene spanning the IRb/SSC boundary exhibited the greatest length among the examined taxa (1,499 bp). Furthermore, the IR regions of *V. keteleeri* ‘Sterile’ were the longest (26,558 bp), whereas the SSC region was the shortest (18,199 bp) among the analyzed species (**Supplementary Figure 2**). These structural features contributed to its genomic distinctiveness within the genus.

**Figure 6:**
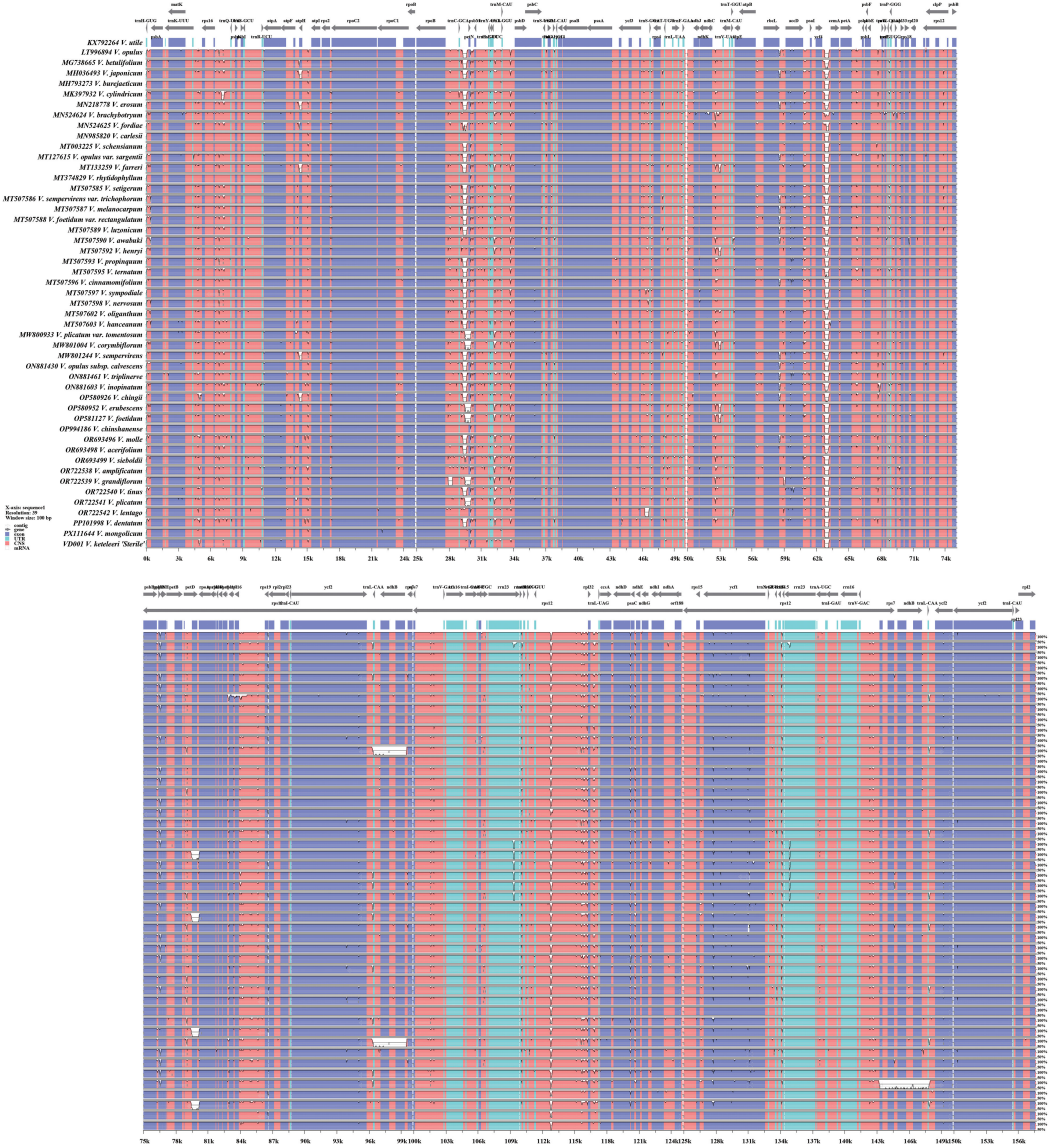
mVISTA-based comparative analysis of chloroplast genome sequences of *Viburnum* species.

Comparative analysis of 49 chloroplast genomes demonstrated high sequence conservation throughout the genus (**Figure 6**). Coding regions were generally more conserved than intergenic spacers. Several highly variable regions were identified, including *trnC*^GCA^-*petN-*p*sbM*, t*rnD-trnY-trnA-trnM*^CAU^-*psbD*, *ycf4-cemA*, and *trnN*^GUU^-*rpl32*-*trnL*^UAG^. Among these regions, *trnC*^GCA^-*petN* and *trnN*^GUU^-*rpl32-trnL*^UAG^ exhibited pronounced sequence divergence in *V. keteleeri* ‘Sterile’ compared with most other *Viburnum* species, indicating their potential utility as candidate molecular markers for species discrimination. Finally, comparative analyses identified 293 lineage-specific single nucleotide polymorphism (SNP) loci in the chloroplast genome of *V. keteleeri* ‘Sterile’ and *V. keteleeri* (**Supplementary Table 1**).

## 4 Discussion

### 4.1 Phylogenetic implications of chloroplast genomes for the taxonomic placement of *V. keteleeri* ‘Sterile’ and *V. keteleeri*

The taxonomic status of *V. keteleeri* ‘Sterile’ and *V. keteleeri* has long been controversial, as morphological traits alone often provide insufficient evidence to distinguish horticultural variants from naturally occurring taxa (Editorial Committee of the Flora of China CAoS, 2011; Clement and Donoghue, 2012). In the present study, phylogenetic analyses based on complete chloroplast genome sequences consistently recovered *V. keteleeri* ‘Sterile’ and *V. keteleeri* within the same terminal clade, supported by extremely short branch lengths and minimal plastome divergence. These results indicate a very close maternal relationship between the two taxa. Previous phylogenomic studies of *Viburnum* have demonstrated that complete chloroplast genomes substantially improve phylogenetic resolution compared with traditional DNA barcodes and a limited number of plastid loci. Ran et al. (2020) analyzed 21 Chinese *Viburnum* species and showed that plastome-scale datasets effectively resolved several previously ambiguous relationships within the genus (Ran et al., 2020). Our findings are consistent with their conclusion that complete chloroplast genomes provide strong phylogenetic signals for resolving relationships among closely related *Viburnum* taxa.

The close phylogenetic affinity observed between *V. keteleeri* ‘Sterile’ and *V. keteleeri* supports the hypothesis that the former originated through artificial selection and domestication from the latter rather than representing an independent evolutionary lineage. Taxonomic uncertainty between horticultural cultivars and their wild progenitor species is not unique to *Viburnum*, but is also common in other ornamental plants subjected to long-term artificial selection. For example, *Camellia japonica* comprises thousands of cultivated forms exhibiting remarkable variation in floral morphology, which has complicated the taxonomic delimitation of cultivars and wild germplasm (Cai et al., 2025). Likewise, *Prunus mume* has undergone centuries of domestication and selection, resulting in hundreds of ornamental cultivars with striking phenotypic diversity despite relatively limited genetic differentiation (Wang et al., 2024). Similar challenges have also been documented in cultivated chrysanthemums (*Chrysanthemum × morifolium*), whose complex hybrid origin and extensive breeding history have obscured relationships between cultivated forms and their wild progenitors (Yue-Ping Ma and Jun, 2020). These examples demonstrate that conspicuous morphological divergence does not necessarily reflect species-level differentiation. Therefore, comparative plastome analysis, as employed in the present study, provides an effective genomic framework for evaluating taxonomic boundaries between horticultural cultivars and their progenitor taxa. Although plastome evidence should be interpreted together with morphological and nuclear genomic data, this integrative approach offers a valuable reference for resolving similar taxonomic controversies across diverse horticultural plant lineages.

### 4.2 Comparative plastome characteristics and their taxonomic significance

Comparative analyses revealed that the chloroplast genomes of *V. keteleeri* ‘Sterile’ and *V. keteleeri* exhibit the typical quadripartite structure found in angiosperms, consisting of LSC, SSC, and two IR regions (Jansen et al., 2005; Ran et al., 2020; Zhao et al., 2020; Gu et al., 2021; Wang et al., 2021; Zhu et al., 2023). The overall genome size, gene content, and genome organization are highly similar to those reported for other *Viburnum* species, including *V. japonicum*, *V. schensianum*, *V. dilatatum*, and *V. sargentii*. Despite this high degree of conservation, several distinctive genomic characteristics were identified, particularly in *V. keteleeri* ‘Sterile’ and *V. keteleeri*, including expanded IR regions, relatively contracted SSC regions, and variation in the position and length of the boundary-associated gene *ycf1* (Guo et al., 2021; Shen et al., 2025). The structural features observed in this study therefore represent potentially informative genomic characters that may assist in distinguishing *V. keteleeri* ‘Sterile’ and *V. keteleeri* from related taxa. In addition, the distribution of SSR loci and codon usage patterns revealed a pronounced A/T bias, a feature commonly reported in chloroplast genomes of flowering plants (Morton, 2022). Such conserved patterns suggest that the plastomes of *V. keteleeri* ‘Sterile’ and *V. keteleeri* have undergone strong evolutionary constraints while retaining lineage-specific variations useful for comparative genomic analyses.

### 4.3 Potential of hypervariable regions and SNP markers for molecular identification

Accurate identification of horticultural cultivars and closely related species remains a major challenge in *Viburnum* taxonomy. Although complete chloroplast genomes provide high discriminatory power, their routine application is limited by sequencing costs and analytical requirements. Therefore, identifying diagnostic molecular markers is essential for practical applications. In this study, a total of 293 lineage-specific SNP loci were identified. Furthermore, several hypervariable regions, including *trnC*^GCA^-*petN-psbM*, *ycf4-cemA*, and *trnN*^GUU^-*rpl32-trnL*^UAG^, exhibited elevated sequence divergence among *Viburnum* species. Our study also identified a stable nucleotide variation between the chloroplast genomes of *V. keteleeri* ‘Sterile’ and *V. keteleeri*. Similar studies have demonstrated that highly variable plastid regions can serve as effective molecular markers for species identification, phylogeographic investigations, and germplasm evaluation (Li et al., 2015; Liu et al., 2024; Liu et al., 2025b). The identification of these candidate loci provides a foundation for the development of cost-effective PCR-based markers. Moreover, designing universal primers based on the conserved regions within the chloroplast genomes of the genus *Viburnum* is not difficult. Such markers could be applied in germplasm conservation, nursery stock certification, cultivar authentication, and intellectual property protection of ornamental plant resources. Future validation using expanded population sampling will be necessary to evaluate marker stability and discriminatory power across diverse geographic regions.

## 5 Conclusions

This study represents a comprehensive investigation of the chloroplast genome characteristics, phylogenetic relationships, and molecular identification potential of *V. keteleeri* ‘Sterile’ and *V. keteleeri*. The complete chloroplast genomes exhibited a typical angiosperm plastome structure with a length of 158,662 bp and contained 133 annotated genes. Comparative genomic analyses revealed a highly conserved genome organization among *Viburnum* species, while several lineage-specific structural features, including IR/SSC boundary variation and distinctive *ycf1* characteristics, were identified. Phylogenetic analyses based on complete chloroplast genome sequences consistently placed *V. keteleeri* ‘Sterile’ together with *V. keteleeri* in a strongly supported terminal clade, indicating an extremely close evolutionary relationship. These findings support the view that *V. keteleeri* ‘Sterile’ most likely represents a horticultural derivative of *V. keteleeri* rather than an independently evolved species. Furthermore, comparative plastome analyses identified multiple hypervariable regions, and 293 candidate lineage-specific SNP loci, which may serve as valuable molecular resources for future marker development. Collectively, these results provide new genomic evidence for clarifying the taxonomic status of *V. keteleeri* ‘Sterile’ and *V. keteleeri*, and establish a foundation for molecular identification, germplasm conservation, breeding utilization, and cultivar protection of *V. keteleeri* ‘Sterile’ and *V. keteleeri*.

## Data Availability

The complete chloroplast genome sequences of Viburnum keteleeri 'Sterile' and Viburnum keteleeri generated in this study have been deposited in the GenBase database of the China National Center for Bioinformation (CNCB-NGDC) and is accessible via the link: https://ngdc.cncb.ac.cn/genbase/review/ebe55e4157c2.

## Supplementary material

Supplementary Figure 1 Single nucleotide polymorphism (SNP) detected between *V. keteleeri* ‘Sterile’ **(A)** and *V. keteleeri* **(B)**.

Supplementary Figure 2 IR scope analysis of the inverted repeat (IR) boundary regions in *Viburnum* species.

Supplementary Figure 3 Validation of the diagnostic nucleotide variation between *Viburnum keteleeri* and *V. keteleeri* ‘Sterile’ using Sanger sequencing chromatograms. Sanger sequencing chromatograms showing the nucleotide pattern at the diagnostic chloroplast position identified between *V. keteleeri* and *V. keteleeri* ‘Sterile’. Samples 1–3 represent independent individuals of *V. keteleeri*, which exhibited an A/C polymorphic pattern at this position, with A as the predominant signal and C as the secondary signal. Samples 4–6 represent independent individuals of *V. keteleeri* ‘Sterile’, which showed a dominant A signal without a comparable secondary C peak.
